# Aqueous deficiency is a contributor to evaporation-related dry eye disease

**DOI:** 10.1186/s40662-019-0172-z

**Published:** 2020-02-01

**Authors:** Charles W. McMonnies

**Affiliations:** Honorary Professor, School of Optometry and Vision Science, University of New South Wales, 77 Cliff Avenue, Northbridge, Sydney, Kensington, New South Wales 2052 Australia

**Keywords:** Aqueous deficient, Evaporative, Dry eyes, Tear break-up

## Abstract

Dry eye disease aetiologies can be classified dichotomously into aqueous deficient and evaporative types although many cases involve combinations of both. Differential diagnosis can be confounded by some features of dry eye disease being common to both aetiologies. For example, short tear break-up times are prime diagnostic findings of tear instability due to lipid and/or mucin deficiencies, but thin tear layers in aqueous deficient eyes also shorten tear break-up times, even at normal range rates of evaporation in eyes without lipid and/or mucin deficiencies. Because tear instability and short tear film break-up times due to thin tear layers can be independent of lipid and/or mucin deficiency, aqueous deficiency can be another form of evaporation-related dry eye. Conversely, tear layers which are thickened by punctal occlusion can be less susceptible to tear break-up. An inflamed lacrimal gland producing reduced quantities of warmer tears can be a basis for thin tear layers and tear instability demonstrated by shorter tear break-up times. Commonly used clinical tests for aqueous deficiency can be unreliable and less sensitive. Consequently, failure to detect or confirm aqueous deficiency as a contributor to short tear break-up times could result in too much weight being given to a diagnosis of meibomian gland deficiency. Less successful treatment outcomes may be a consequence of failing to detect aqueous deficiency. Refining disease classification by considering aqueous deficiency as a contributor to, or even a form of evaporation-related dry eye, could be the basis for more comprehensive and appropriate treatment strategies. For example, some treatment methods for evaporation-related dry eye might be appropriate for aqueous and mucin-deficient as well as lipid-deficient dry eyes. Anti-inflammatory treatment for the lacrimal gland as well as the conjunctiva, may result in increased aqueous production, reduced tear temperature, tear instability and evaporation rates as well as lower osmolarity.

## Background

Accurate diagnosis and classification of dry eye disease is challenging but is necessary as the basis for the provision of the most appropriate therapy [1]. The predominant aetiologies of dry eye disease are aqueous deficient dry eye (ADDE) and evaporative dry eye (EDE) or a combination of them, with or without other etiological factors for dry eye disease [2]. Meibomian gland dysfunction as a contributor to EDE is considered the leading cause of dry eye disease in clinic and population-based studies [2]. For example, of 224 subjects diagnosed with dry eye disease using an objective composite disease severity scale, 49.7% were further classified as having only meibomian gland dysfunction, 14.5% as being purely aqueous deficient, and 35.9% as showing evidence of both meibomian gland dysfunction and ADDE (35.9%) [3]. That 85.6% had at least some evidence of meibomian gland dysfunction [3] explains the common need to provide treatments for lipid deficiencies [1, 2]. The unifying characteristic of dry eye disease is loss of tear homeostasis which can arise from a multitude of factors encompassing eyelid and blink abnormalities in addition to ocular surface or tear component anomalies such as deficiencies in aqueous, lipid or mucin [2]. Apart from tear deficiencies and blink abnormalities, additional pathogenetic factors for dry eye disease include preservatives in topical ophthalmic medications, contact lens wear and ocular surface diseases such as allergy [4].

## Main text

The aqueous layer is secreted by the main and accessory lacrimal glands. In addition, corneal epithelial cells secrete electrolytes and water [5] and conjunctival blood vessels could also leak water, electrolytes and plasma proteins into tears [5]. Kim and co-authors estimated that the mean flow rate from the palpebral lobe of the lacrimal gland to be 0.45 μl/min in a dry eye group (low Schirmer 1 and tear film break-up scores) which is almost exactly 50% of the 0.91 μl/min rate found in a healthy subject group [6]. The mean flow from the Wolfring accessory lacrimal glands was only 1.5 and 1.0% of that found from the main lacrimal gland for dry eye and normal subjects, respectively [6]. Apparently, accessory lacrimal gland aqueous production cannot adequately compensate for lacrimal gland insufficiency [7]. Low level accessory gland contributions appear to explain cases in which dry eye disease developed following unilateral palpebral dacryoadenectomy, while the contralateral eye maintained normal tear functions [7].

### Inflammation

There is now sufficient evidence suggesting that dry eye disease development is the result of cytokine and receptor-mediated inflammatory processes affecting both the lacrimal gland and the ocular surface [8]. Lacrimal gland pathology and inflammation result in ADDE with associated increased tear temperature and osmolarity due to insufficient production [9, 10]. ADDE is exacerbated by even normal rates of evaporation of the aqueous tear phase leading to ocular surface exposure to even greater osmolarity which is a key step in the vicious circle of dry eye disease pathology [10]. The amplifying nature of dry eye disease signs and symptoms toward the end of the day [11] appears to be at least partly in response to tear temperature elevation associated with increasing conjunctival inflammation and tear temperature which progressively accelerates the rate of evaporation throughout the day [12].

### Tear instability

Tear stability is dependent on adequate mucin contributions to a low surface tension with tear film break-up time negatively correlated with surface tension [13]. Tear instability due to mucin deficiency is directly related to chronic inflammation and surface cell apoptosis which is subsequent to cell hyperosmolarity and related goblet cell dysfunction [14]. Consequently, although chronic conjunctival inflammation may be an indication of mucin deficiency due to goblet cell loss and/or dysfunction, a lack of more sensitive and convenient clinical tests for mucin quality and quantity may result in a de-emphasis of these forms of tear deficiency in the diagnosis and classification of dry eye disease [15, 16]. The TFOS DEWS II dichotomous aetiology of dry eye disease classification recognizes the current understanding that an evaporative component to dry eye disease is more common than an ADDE component [2]. The predominance of an evaporative form of dry eye disease [3] which is due to lipid and/or mucin deficiencies may disregard the contributions from ADDE to shorter tear break-up times which are the subject of this review. PubMed searches (11th April 2019) using the terms dry eye and aqueous deficiency; and evaporation; and tear break-up time yielded 177, 243, and 1233 potentially useful publications, respectively. Selections of those which were found to be the most relevant and representative of a balanced account of this topic as well as selected reports referenced in those publications, were included in this review.

### The prevalence and significance of thin tear layers

Even when tears are healthy, evaporation occurs during a normal range inter-blink interval [17–19]. Tear evaporation rates were 5.8 ± 2.7 (10^-7^) g/cm [2] per second for subjects with obstructive meibomian gland dysfunction compared to 4.1 ± 1.4 (10^-7^) g/cm [2] per second for normal controls [20]. These findings indicate that the mean evaporation rate in normal controls was 70.7% of the mean evaporative rate measured in patients with lipid deficiency related to obstructive meibomian gland dysfunction [20]. Consequently, thin tear layers in aqueous deficient eyes can be susceptible to rapid break-up during a normal range inter-blink interval because their thickness can be reduced to a critically thin (break-up) level by even normal range evaporation rates [12, 21]. That even normal eyes could be susceptible to EDE is shown by the finding that the evaporative contribution to aqueous tear loss for normal controls increased from a rate of 23.5 to 41.7% (i.e. an increase by 78.7%) when relative humidity was reduced from 45 to 20% [22]. Apart from lower humidity, higher air temperatures and/or movement as well as lower blink rates and/or higher rates of incomplete blinking can also increase the significance of evaporative loss for normal as well as thin tear layers. Hence, short tear film break-up time findings do not necessarily indicate lipid and/or mucin deficiencies because rapid tear film break-up may also be due to many of these factors including ADDE and an associated thin tear layer. The expectation that ADDE and thinner tear layers can contribute to short tear film break-up times was demonstrated when ADDE and EDE subjects were found to have mean tear break-up times of 4.2 ± 3.9 and 4.4 ± 3.7 s, respectively [3]. Hosaka et al. classified dry eye subjects as ADDE if their Schirmer 1 and tear film break-up times were less than 5 mm and 5 s, respectively [23]. They found that interferometry measurements recorded 5 s after a complete blink for both ADDE (mean age 62.2 ± 11.4 years) and control non-dry eye disease subjects (mean age 27 ± 6.1 years) indicated mean aqueous layer thicknesses of 2.0 ± 1.5 μm and 6.0 ± 2.4 μm, respectively [23]. Loss of 2 μm of tear layer thickness by evaporation during a normal range interblink interval could be of no consequence in eyes with a 6 μm tear layer thickness. However, in ADDE eyes a loss of 2 μm of tear layer thickness by evaporation could too easily cause the cornea to be subjected to extreme hyperosmolar conditions, especially when tear layer thickness is less than 2 μm and the area of tear break-up is greater. The susceptibility of ADDE to evaporation-related pathology was indicated in the Hosaka et al. study by the findings of tear film break-up times being 1.6 ± 1.0 and 8.3 ± 2.5 s for ADDEs and normal control eyes, respectively [23], thus confirming the contribution to tear instability and faster evaporative depletion from ADDE. Apart from pathological lacrimal gland changes such as lymphocytic infiltration in Sjogren’s and non-Sjogren’s ADDE, there are also normal age-related changes with the most common also being inflammatory [9]. Increased flow of blood and heat to an inflamed lacrimal gland appears likely to result in tears which are warmer and so more susceptible to evaporation being delivered to the ocular surface [12]. A reduced quantity of tears being delivered by the inflamed lacrimal gland is also likely to be hyperosmotic [9, 10]. At normal evaporation rates (in the absence of a lipid and/or mucin deficiency), a thin ADDE tear layer has the potential for tear break-up and to develop even greater hyperosmolarity much faster than in normal eyes. In such cases of ADDE, tear instability and tear break-up times could be similar to those found in cases of EDE due to lipid and/or mucin deficiency. For example, Begley and co-authors reported shorter tear film break-up times in association with lower Schirmer 1 findings [11] so that ADDE can be a contributor to EDE which is due to lipid and/or mucin deficiency [2, 12]. However, because thin tear layers shorten tear film break-up time, they increase the chance of making a diagnosis of EDE which may only be partially due to contributions from lipid and/or mucin deficiency. Failure to consider a role for ADDE as a contributor to EDE may lead to overdependence on the treatment of meibomian gland dysfunction for example and the possibility of less than satisfactory outcomes because of aqueous deficiency remaining undiagnosed and untreated. Symptoms which are responses to adverse ambient wind, temperature and/or humidity conditions are consistent with an evaporation-related classification but, apart from lipid and/or mucin deficient tears, thin ADDE tear layers may similarly increase susceptibility to such adverse conditions. In addition, cognitively and/or visually demanding visual tasks which reduce blink frequency and completeness can become too challenging for even stable healthy tears, although even more challenging when tear layers are thin.

### SJOGREN’S and other aqueous deficient syndromes

Sjogren’s syndrome is a chronic systemic autoimmune disease characterized by lymphocytic infiltration of exocrine glands such as the lacrimal glands [24]. As one form of ADDE, the TFOS DEWS II review summarized that only 10% of patients with significant ADDE are likely to have Sjogren’s syndrome although 85% of Sjogren’s syndrome patients report symptoms of dry eye [25]. The prevalence of Sjogren’s syndrome varies widely around the world [26] as well as with variable criteria used for its classification [27]. A meta-analysis of 21 population-based epidemiological studies of primary Sjogren’s syndrome completed in different parts of the world indicated an overall prevalence rate of 0.06% or 61 cases (range 44 to 78) per 100,000 inhabitants [26]. These findings are underestimations to the extent that it is not uncommon for there to be a delay of 5 to 10 years between symptom onset and a diagnosis of Sjogren’s syndrome being made [27]. There would also be cases which are never diagnosed. Even so, that 14.5% of a sample of dry eye disease patients were classified as being purely ADDE [3] indicates that there are many forms of lacrimal gland dysfunction and ADDE which are unrelated to primary Sjogren’s syndrome. The female/male ratio in incidence of primary Sjogren’s syndrome was a mean of 9:1 (range 7.3:1 to 15.6:1) which is consistent with the potential role of sex, sex steroids and other hormones in the pathogenesis of ADDE syndromes [28].

Compared with younger populations, the prevalence of primary Sjogren’s syndrome in the elderly population is between 5 to 8 times higher [24]. Consistent with those findings Obata noted from the examination of post-mortem human lacrimal glands, diffuse fibrosis and atrophy in the orbital lobes which were age-related and predominantly in women [29]. The diagnostic differentiation between primary Sjogren’s syndrome, Sjogren’s syndrome associated with systemic autoimmune diseases, and Sjogren’s syndrome-like presentations of some other systemic autoimmune diseases is difficult [30]. The influence of aging appears to further complicate the differential diagnosis of different classes of ADDE. The prevalence of secondary Sjogren’s syndrome is wide-ranging and varies from 6.5 to 19% depending in part on diagnostic criteria [24]. Pro-inflammatory cytokines can alter both the neural and hormonal support of lacrimal gland function [28]. For example, apart from Sjogren’s syndrome, the lacrimal gland can become a target of the immune system and inflammation in several other diseases such as graft versus host disease following bone marrow transplantation and in cases of sarcoidosis as well as perhaps less commonly in hepatitis C, acquired immunodeficiency syndrome, thyroid disease, diabetes, and even simply, and perhaps more commonly as a result of aging [27].

### Treatment strategies based on more refined disease classifications

The management of dry eye disease requires the use of tear substitutes to protect the ocular surface from desiccation-related damage [31] and they have been a common avenue for treatment [32]. Treatment for lacrimal gland and ocular surface inflammation appear to be key considerations for ADDE-related EDE management [33] Tear production may be increased and tear temperature may be lowered by reducing lacrimal gland inflammation [34]. By this means, the rate of evaporative tear depletion and associated increase in hyperosmolarity which is a stimulus for conjunctival inflammation, could be reduced.^35,36.^ Punctal occlusion to improve tear retention is used in ADDE although tear retention may prolong the presence of pro-inflammatory cytokines on the ocular surface in which case, reducing inflammation may be an important consideration before moving to punctal occlusion [35]. Anti-inflammatory treatment for dry eye disease has involved immunomodulatory agents such as cyclosporine, corticosteroids, tetracycline derivatives and macrolides [36] as well as non-steroidal anti-inflammatory agents and a dietary emphasis on essential fatty acids [37, 38]. Lifitegrast, which is another immunomodulatory drug, has more recently been added to this list [39].

While topical-inflammatory drug access to the ocular surface is direct, access to the lacrimal gland is problematic [40]. More frequent dosing may be beneficial in topical treatment for lacrimal gland inflammation in ADDE [40]. Apart from the risk of difficulties associated with topical administration however, more frequent topical instillation to achieve better access to the lacrimal gland [40] raises the possibility of over-correcting an ocular surface imbalance of pro-inflammatory over anti-inflammatory cytokines. The problems associated with accessing the lacrimal gland with topical therapy [40] are illustrated by a study involving New Zealand white rabbits and treatment with an aqueous nanomicellar Cyclosporine solution (OTX 101 0.05%) [41]. That study found that after instillation of a single topical drop, the maximum lacrimal gland concentration of Cyclosporine was only 2.7, 2.5 and 1.8% of the corneal, superior bulbar conjunctival and third eyelid concentrations, respectively [41]. However, it is possible that more effective access to the lacrimal glands might be achieved with transdermal application of an anti-inflammatory cream, gel or ointment to the skin over them. While any difficulties associated with topical anti-inflammatory treatment could be expected to exacerbate non-adherence to recommended multi-dosage, transdermal therapy may reduce or even avoid some of those kinds of adherence problems.

Improved mucin and lipid functions because of anti-inflammatory treatment to reduce conjunctival and meibomian gland inflammation are suggested by one of the findings from anti-inflammatory treatment for dry eye which was a significant increase in goblet cell numbers [42]. Treatment which reduces lacrimal gland inflammation as a basis for improving aqueous production might also be associated with down-stream consequences due to healthier tears being delivered to the ocular surface which contribute to reduced ocular surface inflammation. However, for many patients, specific treatments for any lipid and/or mucin deficiencies are likely to be indicated in addition to anti-inflammatory approaches. It seems to be the case that ADDE alone can be a possible contributor to evaporative problems, and thus treatments which are otherwise thought to be appropriate for one type of dry eye may be found to be similarly effective for other types. For example, exercises to increase blink rates and reduce incomplete blink rates could help compensate for evaporative tear loss in ADDE as well as in EDE by reducing ocular surface exposure to evaporation [43] and tear conservation using moisture chamber spectacles and humidifiers to slow evaporation [35] may be effective for any form of evaporation-related problem.

## Discussion

Susceptibility to dry eye disease symptoms may be increased according to the degree that lacrimal gland inflammation results in a reduced volume of warmer and hyperosmotic tears being thinly delivered to the ocular surface with an associated amplified rate toward evaporative depletion [12]. ADDE patients have greater risk of evaporation-related dry eye if tear stability is further reduced by lipid and/or mucin deficiencies just as patients with lipid and/or mucin deficiencies can have tear instability and EDE exacerbated by ADDE. Isreb and co-authors reported a correlation between Schirmer 2 ADDE and shorter fluorescein tear break-up in patients with symptoms of dry eye disease and concluded that aqueous and lipid layer deficiencies are not mutually exclusive [21]. Both are contributors to evaporation-related dry eye which is characterized by short tear film break-up times. This review finds that rather than ADDE being just associated with evaporation-related dry eye, it contributes directly to EDE by reducing tear film break-up time. The conclusion that a finding of meibomian gland dysfunction and a lower tear lipid layer thickness is a reliable basis for the correct classification of EDE [21] appears to ignore the possibility that EDE and associated short tear film break-up times may also be partly due to ADDE and associated faster evaporative depletion of a thin tear layer. Even normal evaporation rates appear to be a tear stability challenge for thin tear layers, especially when they are exposed to adverse ambient conditions and/or inefficient blinking for example. In the absence of lipid and mucin deficiencies, ADDE could present as a form of EDE. It appears to be appropriate to suggest that the confidence with which a diagnosis of EDE due to lipid deficiency can be made will depend on the degree to which ADDE or mucin deficiency is also demonstrated. Ideally, diagnostic testing would be able to more confidently differentially diagnose different aetiologies of tear deficiency.

In an 11-year follow-up study of dry eye disease incidence, the most common sign was short fluorescein tear film break-up time which at 47.9% was the only sign to increase in incidence over the 11-year period [44]. Millan and co-authors commented that this finding might indicate a high incidence of meibomian gland dysfunction [44]. However, because they found an increased incidence of autoimmune diseases [44], it is also possible that lacrimal gland inflammation also contributed to shorter tear film break-up times due to more rapid evaporation of warmer and thinner tear layers. Apart from autoimmune diseases, the effects of aging-related lacrimal gland inflammation over 11 years may also have contributed to the findings of Millan and co-authors. As mucin deficiency is difficult to detect clinically [16] and because Schirmer I [45], Schirmer II [46] and Phenol Red Thread [47, 48] tests for ADDE can produce equivocal results [45–48] with associated difficulty diagnosing ADDE, a finding of short tear film break-up time may too easily be attributed to meibomian gland dysfunction. Subsequent emphasis on treatment for meibomian gland dysfunction may be less than successful according to the degree that ADDE has contributed to the short tear film break-up time findings. However, when combined with treatment for ADDE, treatment for any degree of meibomian gland dysfunction might be more successful.

## Conclusions

Refining the diagnostic and classification accuracy for dry eye disease should be the basis for more appropriate treatment strategies [1]. Ideally, evaporation-related dry eye might be better classified in terms of aqueous and/or lipid and/or mucin deficiencies. Since it seems to be the case that ADDE alone can be a contributor to EDE, some treatments which might otherwise be regarded as more appropriate for EDE may be found to also be effective for the treatment of ADDE. Additionally, and notwithstanding the risk of increasing the concentration of inflammatory mediators on the ocular surface, punctal occlusion to thicken tear layer thickness can reduce its susceptibility to tear break-up. The dichotomous ADDE and EDE classifications for dry eye disease aetiology do not appear to be appropriate when making treatment decisions to the extent that tear instability, as indicated by short tear film break-up times, is a feature of aqueous and mucin as well as meibomian gland dysfunction. ADDE patients are at risk for evaporation-related problems although more so if, as well as being a consequence of thin tear layers, tear stability is also reduced by lipid and/or mucin deficiencies.

## Data Availability

Not applicable.
